# Functional Outcomes among a Cohort of Children in Northeastern Brazil Meeting Criteria for Follow-Up of Congenital Zika Virus Infection

**DOI:** 10.4269/ajtmh.19-0961

**Published:** 2020-03-30

**Authors:** Jeanne Bertolli, Jacob Elijah Attell, Charles Rose, Cynthia A. Moore, Flávio Melo, Jennifer Erin Staples, Kim Kotzky, Nevin Krishna, Ashley Satterfield-Nash, Isabela Ornelas Pereira, André Pessoa, Donna Camille Smith, Ana Carolina Faria e Silva Santelli, Coleen A. Boyle, Georgina Peacock

**Affiliations:** 1 Division of Human Development and Disability, Centers for Disease Control and Prevention (CDC), Atlanta, Georgia;; 2 Eagle Global Scientific, San Antonio, Texas;; 3 National Center on Birth Defects and Developmental Disabilities, CDC, Atlanta, Georgia;; 4 Hospital Regional de Guarabira/Governo do Estado da Paraíba, João Pessoa, Brazil;; 5 Division of Vector-Borne Diseases, CDC, Fort Collins, Colorado;; 6 Oak Ridge Institute for Science and Education, Oak Ridge, Tennessee;; 7 Office of Public Health Preparedness and Response, CDC, Atlanta, Georgia;; 8 Transmissible Diseases Department (DEVIT), Ministry of Health of Brazil, Brasilia, Brazil;; 9 Hospital Infantil Albert Sabin, Fortaleza, Brazil;; 10 Division of Congenital and Developmental Disorders, CDC, Atlanta, Georgia;; 11 Division of Global HIV and TB, Center for Global Health, CDC, Atlanta, Georgia

## Abstract

Following the large outbreak of Zika virus in the Western Hemisphere, many infants have been born with congenital Zika virus infection. It is important to describe the functional outcomes seen with congenital infections to allow for their recognition and appropriate interventions. We evaluated 120 children conceived during the 2015–2016 Zika virus outbreak in Paraíba, Brazil, who were approximately 24 months old, to assess functional outcomes. All children met either anthropometric criteria or laboratory criteria suggestive of possible congenital Zika virus infection. We collected results of previous medical evaluations, interviewed parents, and performed physical examinations and functional assessments, for example, the Hammersmith Infant Neurological Examination (HINE). We compared patterns of neurologic outcomes and developmental delay at age 24 months by whether children met anthropometric or laboratory criteria, or both. Among children meeting both criteria, 60% (26/43) were multiply affected (had severe motor impairment, severe developmental delay, and suboptimal HINE scores), compared with 5% (3/57) meeting only laboratory criteria and none (0/20) meeting only anthropometric criteria. Of the remaining 91 children, 49% (45) had developmental delay, with more severe delay seen in children meeting both criteria. Although children meeting physical and laboratory criteria for potential congenital Zika virus infection were more severely affected, we did identify several children with notable adverse neurologic outcomes and developmental delay with no physical findings but potential laboratory evidence of Zika virus infection. Given this, all children who were potentially exposed in utero to Zika virus should be monitored in early childhood for deficits to allow for early intervention.

## INTRODUCTION

Zika virus is believed to have been introduced in Brazil in 2013.^1–3^ However, it was not until August 2015, when healthcare providers in the state of Pernambuco noticed an unusual increase in newborns with microcephaly, that Zika virus was identified to cause congenital infections with serious outcomes.^4^ Although birth defects including microcephaly were the earliest recognized features of congenital Zika virus infection,^5–11^ later findings revealed that the extent of damage caused by intrauterine Zika virus infection is not always apparent at birth^10–12^ but can manifest later,^11,13–15^ raising the possibility that cases could be missed.

The increased incidence of severe microcephaly that first brought Zika virus to attention in Brazil has been likened to “the tip of the iceberg,”^16,17^ yet the size and hallmarks of the rest of the presumed iceberg have been difficult to ascertain in part because of diagnostic challenges.^18^ Aragao et al.^17^ used neuroimaging to describe a spectrum of congenital infections that includes three levels of severity: microcephaly at birth, postnatal microcephaly, and without microcephaly. Other research suggests the possibility of impaired health and development in children without obvious manifestations of congenital infection at birth.^11,12^ Subtle and delayed findings have been identified with congenital Zika virus infection, but their relation to functional deficits is unclear. Few studies have investigated functional outcomes of congenital Zika virus exposure among children aged 12 months or older, born with or without birth defects,^14,19^ and none presented outcomes in relation to infant Zika virus laboratory test results. We followed up a group of infants who were conceived during the 2015–2016 Zika virus outbreak in northeastern Brazil and had been evaluated at 1–7 months of age for evidence of congenital Zika infection^20^ to characterize their functional outcomes at approximately 24 months of age, including neuromotor function, vision, hearing, and developmental delay.

## MATERIALS AND METHODS

### Population.

The Brazilian Ministry of Health, the State Health Secretariat of Paraíba, and the U.S. CDC collaborated on the Zika Outcomes and Development in Infants and Children (ZODIAC) investigation. Infants who were conceived during the 2015–2016 Zika virus outbreak in northeastern Brazil and had participated in a 2016 case–control study to investigate the association between microcephaly and congenital Zika infection^20^ were eligible to participate if they met either laboratory or anthropometric criteria.

1. Laboratory criteria: A nonnegative test for Zika-specific neutralizing antibodies in an infant sample obtained at 1–7 months of age.
2. Anthropometric criteria: Head circumference (HC) ≤ 3rd percentile for gestational age and sex or HC > 3rd percentile for gestational age and sex and HC:body length ≤ 0.65 (i.e., disproportionately small HC, given the length). Note: disproportionate measurements were included as inclusion criteria in the initial case–control study based on early observations of severely impacted infants and later supported by observations of affected fetuses.^21^

We further restricted eligibility for the ZODIAC follow-up investigation to children living in certain areas (i.e., macroregions 1 and 2) of Paraíba state because of logistical constraints. This report includes 19 children with microcephaly and laboratory criteria previously reported by Satterfield-Nash et al.^22^

### Objective.

Our primary objective was to describe the varying severity of functional outcomes that might be associated with congenital Zika virus infection. The protocol for this investigation was approved by the Brazilian National Ethics Committee. The parents who were respondents for this investigation provided informed consent for their own and their child’s participation.

### Assessments.

We analyzed data from three time points, namely, birth, 1–7 months of age, and 19–26 months of age (Table 1). For the investigation at 19–26 months of age, information was collected from medical records, study assessments of the children, and interviews with their caregivers. Information from medical records included HC, length, and weight, reported as *z*-scores and SDs on international growth parameter distributions.^23,24^ The dysmorphologist (CAM) who was on the research team for both the original 2016 case–control study and the ZODIAC study rereviewed photographs of the children to confirm typical Zika phenotypes^8^ or nonspecific dysmorphic features. Licensed physicians performed growth, ophthalmologic, and physical examinations, and a neurologic assessment, and referred children for further evaluation as indicated. Children’s blood was drawn and tested for other factors associated with developmental delay, including blood lead, hematocrit, and thyroid hormone (T4) level.

**Table 1:** Variables, sources, and time frames; Zika Outcomes and Development in Infants and Children investigation, Paraíba, Brazil, 2017

Variable	Source	Child age when assessed
Demographics		
Child	ZODIAC assessment (medical record abstraction)	19–26 months
Child	Case–control records (medical record and assessment)	Birth and 1–7 months
Caregiver	ZODIAC assessment (self-report)	19–26 months
Laboratory evidence of infection		
Child	Case–control (Zika and dengue IgM antibody and neutralization antibody testing of infant specimens)	1–7 months
Medical outcomes		
Dysmorphic features	Case–control (expert clinical evaluation)	1–7 months
Anthropometric parameters	ZODIAC (medical record and assessment) and Case–control records	Birth, 1–7 months, and 19–26 months
Neuromotor function	ZODIAC (physician assessment, Hammersmith Infant Neurological Examination^25^)	19–26 months
Cerebral palsy	ZODIAC (physician assessment)	19–26 months
Hearing impairment	ZODIAC (physician assessment and abstraction of diagnostic evaluation results)	Approximately 19–26 months
Vision impairment (abnormalities of retina and fixation/following)	ZODIAC (physician assessment and functional vision and ophthalmologic examination)	19–26 months
Seizures	ZODIAC assessment (parent report and seizure screener)	19–26 months
Other medical conditions and hospitalization	ZODIAC (medical record abstraction)	≤ 19 months
Developmental delay		
Developmental quotient and developmental age equivalent	ZODIAC assessment (parent report and Ages and Stages Questionnaires, third edition^27^)	19–26 months

ZODIAC = Zika Outcomes and Development in Infants and Children.

Physicians were trained to implement the Hammersmith Infant Neurological Examination (HINE), a standardized neurologic examination that has been validated for ages 3–24 months, to assess neuromotor function and visual and auditory responses.^25^ Trained interviewers administered instruments used to collect caregiver-reported data, including a seizure screener^26^ and the Ages and Stages Questionnaires (ASQ-3),^27^ a series of 21 questionnaires designed to screen the developmental performance of children in five domains (http://agesandstages.com). For severely affected children, we used an amended protocol to administer ASQ-3 questionnaires that were appropriate to a child’s development instead of beginning the assessment with a questionnaire designed for a child of the same biological age (see Supplemental Appendix for details). Field investigators entered data using Research Electronic Data Capture, a secure Web application.^28^

### Analyses.

We compared developmental and neurologic outcomes of children based on whether they met the anthropometric or laboratory criteria of possible congenital infection or both. Children were considered to meet the anthropometric criteria if their HC or HC:length ratio fell within the range described earlier. Children had been tested at the age of 1–7 months for evidence of Zika virus infection as part of the previously conducted case–control study.^20^ Given the timing of sample collection, serologic testing (i.e., Zika virus IgM ELISA and Zika and dengue virus plaque reduction neutralization testing) was performed; no nucleic acid testing was performed on the postnatal samples. Children were considered to meet the laboratory criteria if they had a nonnegative test for Zika neutralizing antibodies (confirmed, presumed, or possible result). For the purpose of this study, Zika virus infection was considered confirmed if an infant blood specimen tested positive for Zika virus–associated IgM antibodies with evidence of neutralizing antibodies against Zika virus. Infection was presumed if an infant sample tested negative for Zika virus–specific IgM antibodies, with Zika virus-dengue virus ratio of maternal–infant neutralizing antibody titers < 1.^20^ Infection was possible if an infant sample tested negative for Zika virus–specific IgM antibodies, with neutralizing antibodies against Zika virus that did not meet the presumed definition.

Developmental delay was measured by the developmental quotient *z*-score (DQz) for each of the five ASQ-3 domains: communication, gross motor skills, fine motor skills, problem solving, and personal–social skills. The developmental quotient (DQ) was defined as the ratio of the child’s developmental (functional) age, determined based on ASQ-3 scores, and the child’s biological age, multiplied by 100. We calculated an ASQ *z*-score for each child on each domain by comparing the child’s score on the questionnaire for that domain to the mean and SD of the distribution of the scores of a large number of Brazilian children, drawn from child daycare centers serving low-income families, who received the same questionnaire.^29^ We used these ASQ *z*-scores to obtain a DQ and a DQz for each child on each domain. Method details are provided in the Supplemental Appendix.

Three developmental delay groups were identified for each of the five ASQ-3 domains, according to children’s ASQ-3 scores and DQz cutoffs: 1) no delay (*z*-score above −1 SD from the mean), 2) possible delay (*z*-score from −1 to −2 SD), and 3) likely delay (*z*-score below −2 SD), according to convention.^27^ For this study, the degree of overall developmental delay incorporating scores on all five ASQ domains was described as follows: 1) none: above −1 SD on all five ASQ-3 domains, 2) severe: below −2 SD on at least two domains, and 3) mild to moderate: the remaining combinations, that is, *z*-scores below −2 SD on one domain or from −1 SD to −2 SD on at least one domain.

Recognizing that some congenitally infected children may have subtle findings that are not distinctive for congenital Zika infection,^14,15,19^ we looked for patterns of functional outcomes by the anthropometric or laboratory criteria, or both. We examined proportions of children with developmental delay (mild to moderate or severe) on any ASQ-3 domain, with and without a neurologic deficit (i.e., severe motor impairment, cerebral palsy of any type, a suboptimal HINE score on any domain, functional vision problems, a positive seizure screen) within and across subgroups (anthropometric, laboratory, or both). To investigate whether the findings varied by strength of laboratory evidence that supported congenital Zika virus infection versus passive maternal transfer of neutralizing antibodies, we further subdivided children and analyzed the outcome data according to their original laboratory classification.^20^ We hypothesized that children with congenital Zika infection might have a pattern of domain-specific developmental delay, so we also examined children with developmental delay in each of the five ASQ-3 domains by the anthropometric and laboratory criteria.

## RESULTS

**Figure 1.**
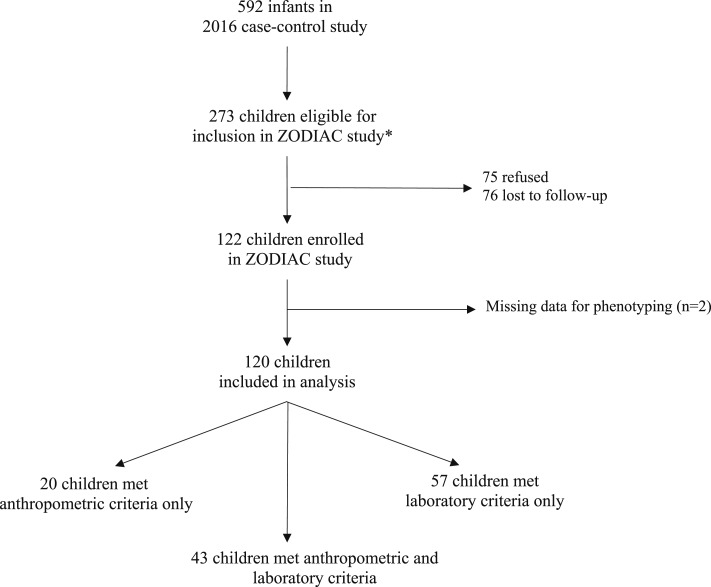
Eligibility for the Zika Outcomes and Development in Infants and Children investigation, Paraíba, Brazil, 2017. Anthropometric criteria: head circumference (HC) ≤ 3rd percentile for gestational age and sex or HC > 3rd percentile for gestational age and sex and HC: body length ≤ 0.65; Laboratory criteria: non-negative Zika neutralizing antibody test results in an infant sample.

Of the 592 children included in the 2016 case–control study, 273 were eligible for follow-up (Figure 1). Overall, 122 children (45%) participated in the follow-up investigation, 76 (28%) were lost to follow-up, and 75 families (28%) refused to participate. Two children were excluded from analysis because they were missing critical data, leaving 120 children who were included based on the anthropometric and/or laboratory criteria and assessed from July to October 2017. At the time of evaluation for the ZODIAC investigation, the median age was 23 months (range, 19–26 months). Table 2 presents demographic information on the children and their caregivers.

**Table 2:** Demographic characteristics of 120 children meeting anthropometric and/or laboratory criteria for follow-up of congenital Zika virus infection, and of their caregivers and families, Paraíba, Brazil, 2017

Characteristic	Number	Percent
Child’s sex		
Male	60	50
Female	60	50
Child’s age at assessment (months)		
19–20	11	9
21–22	49	41
23–24	46	38
25–26	14	12
Caregiver relationship with the child		
Mother	115	96
Other caregiver*	5	4
Caregiver race		
White	14	12
Black	15	13
Brown	81	68
Yellow	8	7
Indigenous	2	2
Caregiver age (years)		
≤ 18	8	7
19–23	29	24
24–28	23	19
29–33	34	28
> 33	26	22
Caregiver education (years)		
≤ 6	24	20
7–8	24	20
9–11	36	30
≥ 12	36	30
Monthly family income (R$)†		
< 500	27	23
500–1,499	69	58
≤ 1,500	18	15
Unknown	6	5
Trouble covering basic expenses		
Never	38	32
Rarely	31	26
Somewhat often	25	21
Very often	25	21
Unknown	1	1
Household size		
2–4	78	65
5–7	37	31
> 8	5	4

* Other caregivers included one aunt, one father, two grandparents, and one godmother.

† R$500.00 = US$154.32 in 2017.

Forty-three (36%) children met both anthropometric and laboratory criteria, including 27 children who had microcephaly and 16 who were classified as disproportionate. Of the remaining children, 57 (48%) met only the laboratory criteria and 20 (17%) met only the anthropometric criteria for inclusion in the follow-up study; all children with only the anthropometric criteria were disproportionate, and none had microcephaly (Table 3).

**Table 3:** Functional outcomes* at approximately 24 months of age, among children meeting anthropometric and/or laboratory criteria for follow-up of congenital Zika virus infection, Paraíba, Brazil, 2017

Outcome	Anthropometric/laboratory criteria† of congenital Zika infection		
	Microcephaly/disproportionate with laboratory criteria§	Disproportionate without laboratory criteria	Laboratory criteria only
	*N* = 43, *N* (%)	*N* = 20,‡ *N* (%)	*N* = 57, *N* (%)
Severe motor impairment	26 (61)	0 (0)	0 (0)
Cerebral palsy	25 (58)	0 (0)	0 (0)
Impaired response to auditory stimuli	23 (54)	0 (0)	0 (0)
Impaired response to visual stimuli—HINE	21 (49)	0 (0)	0 (0)
Suboptimal score on any HINE domain	26 (61)	0 (0)	1 (2)
Developmental delay classification‖ (overall)			
Severe	27 (63)	2 (10)	3 (5)
Mild to moderate	7 (16)	9 (45)	26 (46)
None	9 (21)	9 (45)	28 (49)
Vision			
Retinal abnormalities	7 (16)	0 (0)	0 (0)
Abnormal fixation and following	16 (37)	0 (0)	0 (0)
Positive seizure screen	18 (43)	0 (0)	2 (4)
Missing	1	0	1

HC = head circumference; HINE = Hammersmith Infant Neurological Examination.

* Children were evaluated for functional outcomes, including developmental delay (using the Ages and Stages v.3 [ASQ-3] questionnaires) and neurologic outcomes (using the HINE) at approximately 24 months of age.

† Anthropometric criteria: 1) microcephaly (HC ≤ 3rd percentile for gestational age and sex) or 2) HC > 3rd percentile for gestational age and sex and disproportionate (HC:body length ≤ 0.65); laboratory criteria: nonnegative Zika neutralizing antibody test results.

‡ Three children had missing HC at birth and disproportionate HC:length when measured at 1–7 months of age.

§ In this group, 23 children had microcephaly and were disproportionate (had HC:length ratio ≤ 0.65); 4 children had microcephaly with HC:length ratio > 0.65, and 16 children were disproportionate but did not have microcephaly.

‖ Severe: *z*-scores below −2 SD from the mean on at least two ASQ-3 domains, mild to moderate: *z*-scores below −2 SD on one domain or from −1 SD to −2 SD on at least one domain, none: *z*-scores (all domains) above −1 SD from the mean.

Severe motor impairment, cerebral palsy, HINE scores indicating impaired response to auditory stimuli and impaired response to visual stimuli, a suboptimal score on any HINE domain, and a positive screen for seizures were observed in children meeting both anthropometric and laboratory criteria but not among children meeting only anthropometric or laboratory criteria, with the exception of one child in the latter group, who had suboptimal HINE scores without the other outcomes we investigated (Table 3). Retinal abnormalities were observed in seven children (16%) meeting both criteria compared with none meeting only the anthropometric or laboratory criteria. Also 16 children (37%) meeting both criteria were found to have functional vision impairment compared with none in the other two groups.

Of the children meeting both anthropometric and laboratory criteria, 63% had severe developmental delay, 16% had mild to moderate delay, and 23% had no delay, according to ASQ-3 assessments. Ten percent (two of 20 children) meeting only the anthropometric criteria and 5% (three of 57) meeting only the laboratory criteria had severe developmental delay. Nine (45%) of 20 children meeting only the anthropometric criteria and 24 (42%) of 57 meeting only the laboratory criteria had mild to moderate developmental delay.

Table 4 shows that among 43 children who met both anthropometric and laboratory criteria, 26 (60%) were multiply affected with severe motor impairment, severe developmental delay, and multiple suboptimal HINE scores, with or without vision problems. Of these 26 children, 12 (46%) had laboratory results indicating confirmed Zika virus infection, six (23%) had presumed Zika virus infection, and eight (31%) had possible Zika virus infection. All the children with test results indicating confirmed Zika virus infection had microcephaly. One child with these laboratory findings and disproportionate measurements at birth developed microcephaly postnatally. Among 57 children meeting the laboratory criteria only, three (5%) had adverse neurologic outcomes in addition to developmental delay. One of these children had the same pattern of multiple, severe neurologic outcomes and severe developmental delay described in the multiply affected group meeting both criteria; this child had laboratory results indicating possible infection. The other two children had mild to moderate developmental delay and a positive seizure screen without other adverse neurologic outcomes, and laboratory results indicating possible and presumed infection. None of the 20 children who only had anthropometric features (all with disproportionate HC:length) were multiply affected.

**Table 4:** Neurologic outcomes and developmental delay* at approximately 24 months of age, among children meeting the anthropometric and/or laboratory criteria† of congenital Zika virus infection, Paraíba, Brazil, 2017

Criteria of congenital Zika virus infection	Neurologic outcomes and developmental delay‡	Developmental delay only§	Neither developmental delay nor neurologic outcomes
Microcephaly/disproportionate‖ with laboratory criteria¶ (*N* = 43)			
Total	26 (60)	8 (19)	9 (21)
Confirmed	12	0	0
Presumed	6	1	0
Possible	8	7	9
Laboratory criteria only (*N* = 57)			
Total	3 (5)	26 (46)	28 (49)
Confirmed	0	1	0
Presumed	1	7	5
Possible	2	18	23
Disproportionate without laboratory criteria¶ (*N* = 20)			
No evidence of infection	0	11 (55)	9 (45)
Total	29 (24)	45 (38)	46 (38)

* Children were evaluated for functional outcomes, including developmental delay (using the Ages and Stages v.3 [ASQ-3] questionnaires) and neurologic outcomes (using the Hammersmith Infant Neurological Examination) at approximately 24 months of age.

† Anthropometric criteria: 1) microcephaly (head circumference [HC] ≤ 3rd percentile for gestational age and sex) or 2) disproportionate (HC > 3rd percentile for gestational age and sex and HC:body length ≤ 0.65); laboratory criteria: nonnegative Zika neutralizing antibody test results.

‡ All children in this column in the “microcephaly/disproportionate with laboratory criteria” group and all but two children in this column in the “laboratory criteria only” group had severe motor impairment, severe developmental delay, and multiple suboptimal Hammersmith Infant Neurological Examination scores, with or without vision problems, when evaluated at 24 months of age. The two children in the “laboratory criteria only” group had severe developmental delay and a positive seizure screen, without the other neurologic outcomes.

§ Children in this group had developmental delay on any of the five ASQ-3 domains, as indicated by their ASQ-3 scores and developmental quotient cutoffs, that is, a z-score ≤ −1 SD. For one of eight children with developmental delay only in the “microcephaly/disproportionate with laboratory criteria” group, two of 26 children in the “laboratory criteria only” group, and two of 11 children in the “disproportionate without laboratory evidence” group, developmental delay was severe, that is, *z*-score < −2 SD on at least two domains.

‖ In this group, 23 children had microcephaly and were disproportionate (had HC:length ≤ 0.65); four children had microcephaly with HC:length > 0.65, and 16 children were disproportionate but did not have microcephaly.

¶ For the purpose of this study, a confirmed Zika virus infection was defined as an infant blood specimen testing positive for Zika virus–associated IgM antibodies with evidence of neutralizing antibodies against Zika virus. A presumed infection was defined as an infant sample testing negative for Zika virus–specific IgM antibodies, with Zika virus-dengue virus ratio of maternal–infant neutralizing antibody titers < 1.^20^ A *possible* infection was defined as an infant sample testing negative for Zika virus–specific IgM antibodies, with neutralizing antibodies against Zika virus that did not meet the presumed definition.

Developmental delay without other outcomes was noted for eight children (19%) who met both anthropometric and laboratory criteria (none with laboratory results indicating confirmed infection), compared with 11 (55%) meeting only the anthropometric criteria and 26 (46%) meeting only the laboratory criteria. Of children meeting only the laboratory criteria, 46% (26/57) had ASQ-3 scores, indicating mild to moderate developmental delay, including the only child in the “laboratory criteria only” group whose test results indicated confirmed infection; seven (54%) of 13 with presumed infection; and 18 (42%) of 43 with possible infection (data not shown). Neither developmental delay nor adverse neurologic outcomes were observed for nine (45%) of the children meeting only the anthropometric criteria, compared with 28 (49%) meeting the laboratory criteria only, and nine (21%) meeting both; all nine of the latter group had possible laboratory evidence of infection.

**Figure 2.**
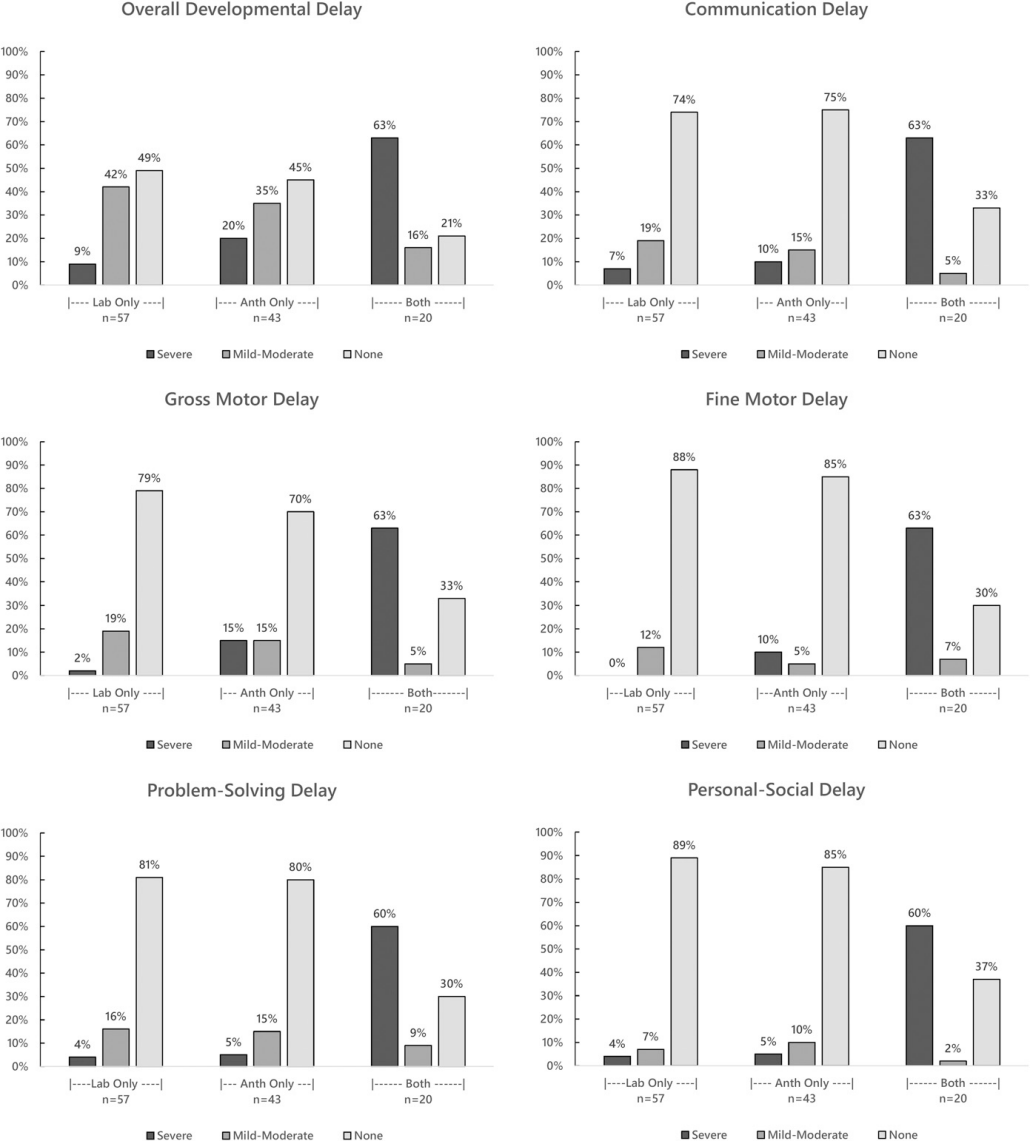
Overall developmental delay and domain-specific delay at approximately 24 months of age, among children meeting the anthropometric and/or laboratory criteria for follow-up of congenital Zika virus infection, Paraíba, Brazil, 2017. Anthropometric (Anth) criteria: head circumference (HC) ≤ 3rd percentile for gestational age and sex or HC > 3rd percentile for gestational age and sex and HC: body length ≤ 0.65; Laboratory (Lab) criteria: non-negative Zika neutralizing antibody test results in an infant sample. Overall development classification: 1) none: z-scores above -1 SD from the mean on all five ASQ-3 domains, 2) severe: below -2 SD on at least 2 domains, and 3) mild-moderate: the remaining combinations, i.e., z-scores below -2 SD on one domain or from -1 SD to -2 SD on at least one domain. Domain-specific development classification: 1) none: z-score above -1 SD from the mean, 2) possible delay (z-score from -1 to -2 SD), and 3) likely delay (z-score below -2 SD).^25^

Domain-specific developmental delay is presented in Figure 2. Children meeting both anthropometric and laboratory criteria were more likely to have overall developmental delay and delay in any specific domain. Similar percentages of children meeting only the laboratory or anthropometric criteria had developmental delay on specific ASQ-3 domains, with the exception of the gross and fine motor domains.

The majority of children in all three categories had a blood lead level above the reference value of > 5 µg/dL: 60% (12 of 20) children meeting the anthropometric criteria only, including six of 11 with developmental delay; 64% (35 of 55) children meeting the laboratory criteria only, including 17 of 29 with developmental delay; and 70% (30 of 43) children meeting both, including 25 of 34 with developmental delay. Hematocrit was below the reference range for six (30%) of the children meeting the anthropometric criteria, nine (16%) meeting the laboratory criteria, and five (12%) meeting both. None of the children in any of the three groups had a free T4 value below the reference range. Among the 74 children who had developmental delay with or without adverse neurologic outcomes, 16 (22%) had nonspecific dysmorphic features that were consistent neither with the recognized congenital Zika phenotype nor with other recognized congenital syndromes.

## DISCUSSION

Follow-up of children who were conceived during a large Zika virus outbreak and who met anthropometric or laboratory criteria suggesting congenital Zika infection revealed heterogeneity of functional outcomes at approximately 24 months of age. Those who had microcephaly or disproportionate measurements and met the laboratory criteria were more likely to have multiple, severe functional impairments, and if severely affected, were more likely to have stronger laboratory evidence of possible congenital Zika virus infection than children who met only the laboratory or anthropometric criteria. The group meeting both criteria had the most recognizable presentation of congenital Zika infection.^5–11^

Other investigators have reported that children with evidence of prenatal exposure to Zika virus and without birth defects typically have a less severe clinical presentation, although some have seizures, abnormal hearing and vision, and other neurodevelopmental abnormalities.^14,15,30,31^ However, most children in these studies did not have all relevant evaluations, so functional deficits and birth defects may have been under-identified. We found that 5% of children without physical findings but with some potential laboratory evidence of Zika virus infection had adverse neurologic outcomes and developmental delay. This included one child with the same pattern of severe developmental delay and multiple adverse neurologic outcomes described in the multiply affected group meeting both anthropometric and laboratory criteria, and two children whose only outcomes were mild to moderate developmental delay and seizures. The strength of laboratory evidence for these cases varies, making the correlation between these findings and congenital Zika virus infection challenging.

Others have reported evidence of modest cognitive deficits in infants exposed to Zika virus prenatally who did not have microcephaly.^14,32^ Consistent with these reports, we identified children who had mild to moderate developmental delay without adverse neurologic outcomes among those meeting only the laboratory criteria. However, only one child in this group had laboratory results that met our study definition of a confirmed Zika virus infection. The majority had laboratory results that precluded definitive interpretation because of the inability to distinguish infection from passive transfer of maternal antibodies. The fact that 37 of 100 children with some laboratory evidence of Zika virus infection in our study had neither developmental delay nor adverse neurologic outcomes suggests that either some children infected with Zika virus (perhaps those infected late in gestation) may not develop the functional outcomes we studied or the neutralizing antibodies detected in the children were only from the maternal transfer of these antibodies.

We also explored the possibility that the observed developmental delay could be attributable to causes other than congenital Zika virus infection. Developmental delay, as measured by the ASQ-3 instruments, was common overall in our study population (noted in 62% of the children, including 55% of those with no laboratory evidence of infection). Developmental delay was more often severe in children with adverse neurologic manifestations identifiable by the HINE. General estimates of the prevalence of developmental delay in the pediatric population range from 5% to 10%,^33^ but the prevalence varies according to case definition and population demographics, including parental education and socioeconomic status.^34,35^

We attempted to adjust for the prevalence of developmental delay by standardizing ASQ-3 scores to the distribution of the scores of Brazilian children from low-income families who received the same questionnaire. The higher prevalence of developmental delay in our study population might in part be explained by the fact that the children we studied were chosen for follow-up because they had anthropometric features or laboratory results that suggested possible congenital Zika infection. We also identified anemia and elevated blood lead levels as possible causes of developmental delay other than possible congenital Zika virus infection in our study population, and the presence of nonspecific dysmorphic features in 22% of the children with developmental delay may indicate a syndromic diagnosis that could not be explored in this investigation; other prenatal infectious or noninfectious exposures could have affected children’s developmental outcomes. Given these potential confounders, it is challenging to know if our results regarding less severe outcomes can be specifically attributed to congenital Zika virus infection.

For children whose only manifestations of congenital infection are developmental delay, early recognition is a challenge. Aragao et al.^17^ used computed tomography (CT) imaging to describe a spectrum of brain damage in congenital Zika infection. However, in many settings, including northeastern Brazil during the 2015–2016 outbreak, imaging may not be readily available, or indicated if available. Furthermore, imaging findings may not be specific. In our study, only 14 of the 43 children with both anthropometric and laboratory evidence of infection had CT imaging results available in medical records. When Zika exposure is suspected, screening for developmental delay, in addition to neuroimaging and HC measurement, may be necessary to identify affected children for early intervention.

Other investigators have documented features that have expanded the characterization of outcomes of congenital Zika infection, including infants who had typical HCs with nonspecific neurologic signs and isolated eye abnormalities.^14,15,17,35^ We did not find, as other researchers did, that eye abnormalities occurred as the primary or only manifestation of congenital Zika infection.^35^ We did, however, identify four children meeting the laboratory criteria who had retinal findings that are considered pathognomonic for congenital Zika virus infection,^36^ although none had test results indicating confirmed Zika virus infection, by the study definition. Without an ophthalmologic examination, the possible contribution of Zika virus to the medical and developmental outcomes in these children could have been missed.

As for other studies of congenital Zika infection, the main limitations of this investigation were the inability to accurately identify infected children or exposure to maternal infection, to rule out other infectious or noninfectious causes of neurologic disorders and developmental delay, and to obtain complete medical histories from available records. Furthermore, serologic testing was performed when the infants were 1–7 months of age and residing in areas where there was Zika virus circulation. When laboratory findings suggested infection, we assumed, but were unable to confirm that infection occurred prenatally. This investigation was also limited by small numbers in several categories. Further evaluation is warranted to assess whether comparing Zika-dengue maternal–infant antibody ratios is a valid method for refining interpretation of Zika laboratory test results. Despite these limitations, this study incrementally advances the state of knowledge about the possible consequences of congenital Zika infection by addressing functional outcomes, including developmental delay.

## CONCLUSION

Identifying infants with congenital Zika virus infection remains challenging. Laboratory confirmation of congenital Zika virus infection may not be possible in all infected infants, and anthropometric characteristics are heterogeneous. Because of this, children with congenital Zika infection without obvious clinical manifestations could be missed. When Zika exposure is suspected, ongoing monitoring during the early childhood period, including screening for developmental delay in addition to neuroimaging and HC measurement, may be necessary to identify affected children for early intervention. Questions remain about how closely prenatally exposed children should be monitored and what clinical evaluations are appropriate.

## Supplemental appendix

Supplemental materials

## References

[1] Faria NR 2016 Zika virus in the Americas: early epidemiological and genetic findings. Science 352: 345–349. 27013429 10.1126/science.aaf5036 PMC4918795

[2] Brito C, 2015 Zika virus: A new chapter in the history of medicine. Acta Med Port 28: 679–680. 26849748 10.20344/amp.7341

[3] Cardoso CW Paploski JAD Kikuti M Rodrigues MS Silva MM Campos GS Sardi SI Kitron U Reis MG Ribeiro GS, 2015 Outbreak of actue exanthematous illness associated with Zika, chikungunya, and dengues viruses, Salvador, Brazil [letter]. Emerg Infect Dis 21: 2274–2276. 26584464 10.3201/eid2112.151167 PMC4672408

[4] Pan American Health Organization, 2016 Timeline of Emergence of Zika Virus in the Americas*.* Available at: http://www.paho.org/hq/index.php?option=com_content&view=article&id=11959:timeline-of-emergence-of-zika-virus-in-the-americas&Itemid=41711&lang=en. Accessed July 15, 2019.

[5] Kleber de Oliveira W Cortez-Escalante J De Oliveira WT do Carmo GM Henriques CM Coelho GE Araújo de França GV, 2016 Increase in reported prevalence of microcephaly in infants born to women living in areas with confirmed Zika virus transmission during the first trimester of pregnancy–Brazil, 2015. MMWR Morb Mortal Wkly Rep 65: 242–247. 26963593 10.15585/mmwr.mm6509e2

[6] Brasil P 2016 Zika virus infection in pregnant women in rio de Janeiro. N Engl J Med 375: 2321–2334. 26943629 10.1056/NEJMoa1602412 PMC5323261

[7] Rasmussen SA Jamieson DJ Honein MA Petersen LR, 2016 Zika virus and birth defects–reviewing the evidence for causality. N Engl J Med 374: 1981–1987. 27074377 10.1056/NEJMsr1604338

[8] Moore CA 2017 Characterizing the pattern of anomalies in congenital Zika syndrome for pediatric clinicians. JAMA Pediatr 171: 288–295. 27812690 10.1001/jamapediatrics.2016.3982 PMC5561417

[9] Schuler-Faccini L Brazilian Medical Genetics Society–Zika Embryopathy Task Force, 2016 Possible association between Zika virus infection and microcephaly–Brazil, 2015. MMWR Morb Mortal Wkly Rep 65: 59–62. 26820244 10.15585/mmwr.mm6503e2

[10] França GV 2016 Congenital Zika virus syndrome in Brazil: a case series of the first 1501 livebirths with complete investigation. Lancet 388: 891–897. 27372398 10.1016/S0140-6736(16)30902-3

[11] van der Linden V 2016 Description of 13 infants born during October 2015-January 2016 with congenital Zika virus infection without microcephaly at birth - Brazil. MMWR Morb Mortal Wkly Rep 65: 1343–1348. 27906905 10.15585/mmwr.mm6547e2

[12] Nogueira ML 2018 Adverse birth outcomes associated with Zika virus exposure during pregnancy in São José do Rio Preto, Brazil. Clin Microbiol Infect 24: 646–652. 29133154 10.1016/j.cmi.2017.11.004

[13] Moura da Silva AA 2016 Early growth and neurologic outcomes of infants with probable congenital Zika virus syndrome. Emerg Infect Dis J 22: 1953–1956. 10.3201/eid2211.160956 PMC5088045 27767931

[14] Nielsen-Saines K 2019 Delayed childhood neurodevelopment and neurosensory alterations in the second year of life in a prospective cohort of ZIKV-exposed children. Nat Med 25: 1213–1217. 31285631 10.1038/s41591-019-0496-1 PMC6689256

[15] Einspieler C The GM Zika Working Group, 2018 Association of infants exposed to prenatal Zika virus infection with their clinical, neurologic, and developmental status evaluated via the general movement assessment tool. JAMA Netw Open 2: e187235. 10.1001/jamanetworkopen.2018.7235 PMC6431234 30657537

[16] Oliveira Melo AS Malinger G Ximenes R Szejnfeld PO Alves Sampaio S Bispo de Filippis AM, 2016 Zika virus intrauterine infection causes fetal brain abnormality and microcephaly: tip of the iceberg? Ultrasound Obstet Gynecol 47: 6–7. 26731034 10.1002/uog.15831

[17] Aragao MFVV 2017 Nonmicrocephalic infants with congenital Zika syndrome suspected only after neuroimaging evaluation compared with those with microcephaly at birth and postnatally: how large is the Zika virus “iceberg”? Am J Neuroradiol 38: 1427–1434. 28522665 10.3174/ajnr.A5216 PMC7959892

[18] Munoz-Jordan JL, 2017 Diagnosis of Zika virus infections: challenges and opportunities. J Infect Dis 216 (Suppl–10): S951–S956. 29267922 10.1093/infdis/jix502 PMC5853979

[19] Wheeler AC Ventura CV Ridenour T Toth D Nobrega LL Silva de Souza Dantas LC Rocha C Bailey DB Jr Ventura LO, 2018 Skills attained by infants with congenital Zika syndrome: pilot data from Brazil. PLoS One 13: e0201495. 30048541 10.1371/journal.pone.0201495 PMC6062124

[20] Krow-Lucal ER Paraíba Microcephaly Work Group, 2018 Association and birth prevalence of microcephaly attributable to Zika virus infection among infants in Paraíba, Brazil, in 2015–16: a case-control study. Lancet Child Adolesc Health 2: 205–213. 30169255 10.1016/S2352-4642(18)30020-8

[21] Walker CL 2018 Femur-sparing pattern of abnormal fetal growth in pregnant women from New York city after maternal Zika virus infection. Am J Obstet Gynecol 219: 187.e1–187.e20. 29738748 10.1016/j.ajog.2018.04.047 PMC6066422

[22] Satterfield-Nash A 2017 Health and development at age 19–24 months of 19 children who were born with microcephaly and laboratory evidence of congenital Zika virus infection during the 2015 Zika virus outbreak–Brazil, 2017. Morb Mortal Wkly Rep 66: 1347–1351. 10.15585/mmwr.mm6649a2 PMC5730218 29240727

[23] International Fetal and Newborn Growth Consortium for the 21st Century, 2017 Standards for Newborns and References for Very Preterm Infants. Available at: https://intergrowth21.tghn.org/. Accessed July 15, 2019.

[24] World Health Organization, 2017 Child Growth Standards. Head Circumference for Age. Available at: http://www.who.int/childgrowth/standards/hc_for_age/en/. Accessed July 15, 2019.

[25] Maitre NL Chorna O Romeo DM Guzzetta A, 2016 Implementation of the Hammersmith infant neurological exam in a high-risk infant follow-up program. Pediatr Neurol 65: 31–38. 27765470 10.1016/j.pediatrneurol.2016.09.010 PMC5395423

[26] Douglass LM 2016 A novel parent questionnaire for the detection of seizures in children. Pediatr Neurol 54: 64–69.e1. 26552646 10.1016/j.pediatrneurol.2015.09.016

[27] Squires J Twombly E Bricker D Potter L, 2009 ASQ-3™ User’s Guide, 3rd edition Baltimore, MD: Paul H. Brooks Publishing Company, Inc., 157–169.

[28] Harris PA Taylor R Thielke R Payne J Gonzalez N Conde JG, 2009 Research electronic data capture (REDCap) – a metadata-driven methodology and workflow process for providing translational research informatics support. J Biomed Inform 42: 377–381. 18929686 10.1016/j.jbi.2008.08.010 PMC2700030

[29] Filgueiras A Pires P Maissonette S Landeira-Fernandez J, 2013 Psychometric properties of the Brazilian-adapted version of the ages and stages questionnaire in public child daycare centers. Early Hum Dev 89: 561–576. 23507472 10.1016/j.earlhumdev.2013.02.005

[30] Lopes Moreira ME 2018 Neurodevelopment in infants exposed to Zika virus in utero. N Engl J Med 379: 2377–2379. 30575464 10.1056/NEJMc1800098 PMC6478167

[31] Rice ME 2018 Vital signs: Zika-associated birth defects and neurodevelopmental abnormalities possibly associated with congenital Zika virus infection–U.S. territories and freely associated states, 2018. MMWR Morb Mortal Wkly Rep 67: 858–867. 30091967 10.15585/mmwr.mm6731e1 PMC6089332

[32] Valdes V 2019 Cognitive development of infants exposed to the Zika virus in Puerto Rico. JAMA Netw Open 2: e1914061. 31651970 10.1001/jamanetworkopen.2019.14061 PMC6822087

[33] Simeonson RJ Sharp MC, 1992 Developmental delays. Hoekelman RA Friedman SB Nelson NM Seidel HM, eds. Primary Pediatric Care, 2nd edition St. Louis, MO: C. V. Mosby, 867–870.

[34] Gottlieb CA Maenner MJ Cappa C Curkin MS, 2009 Child disability screening, nutrition, and early learning in 18 countries with low and middle incomes: data from the third round of UNICEF’s multiple indicator cluster survey (2005–06). Lancet 374: 1831–1839. 19944864 10.1016/S0140-6736(09)61871-7

[35] Durkin M, 2002 The epidemiology of developmental delays in low-income countries. Ment Retard Dev Disabil Res Rev 8: 206–211. 12216065 10.1002/mrdd.10039

[36] Tsui I 2018 Eye findings in infant with suspected or confirmed antenatal Zika virus exposure. Pediatrics 142: e20281104. 10.1542/peds.2018-1104 PMC6317824 30213843

