# Supplementary material for: Functional Outcomes among a Cohort of Children in Northeastern Brazil Meeting Criteria for Follow-Up of Congenital Zika Virus Infection
Source: Am J Trop Med Hyg. 2020 Mar 30;102(5):955–63. doi: 10.4269/ajtmh.19-0961 (PMC7204564; doi:10.4269/ajtmh.19-0961)
Supplement: Supplementary file 1 [file tpmd190961.SD1.pdf]

Supplemental Appendix

The Ages and Stages Questionnaires, third edition (ASQ-3) are developmental screening instruments designed for interviewing parents to determine the need for follow-up of their children or referral for diagnosis and intervention services. The ASQ-3 Questionnaires are routinely used for making decisions regarding monitoring or referral based on a "developmental quotient" (DQ), i.e., a number expressing the development of a child by dividing the age of the group into which the questionnaire response scores place the child by the child's chronological age and multiplying by 100.Typically, the child's parent/caregiver will receive the questionnaire that is appropriate for the child's biological age (i.e., a child at age 6 months would receive the 6-month questionnaire). A clinician might compare the child's score on this questionnaire to assess whether the child appears to be developing on par with other children of the same age, and if not, the extent to which the child is deviating from the expected scores for his/her age, and the age at which the child is functioning.

We used the ASQ-3 Questionnaires in the ZODIAC investigation to evaluate the development of children with anthropometric and laboratory criteria for follow up of congenital Zika virus infection. Because the distribution of ASQ-3 scores for Brazilian children is known to be different from the distribution to which the ASQ tool is normed (Squires, Twombly, Bricker, & Potter, 2009), we incorporated a conversion factor to estimate DQs for the children in our study population. In addition, since some children who participated in the investigation were known to have severe developmental delay, we used an amended protocol to administer the ASQ-3 questionnaire that was appropriate to a child's development, instead of beginning the assessment with the questionnaire typically used for a child of the same age.

Our protocol was as follows:

1) On each of the 5 ASQ-3 domains, caregivers were administered the 6-month questionnaire first.

2) If the caregiver's responses indicated the child did not pass the first two items (those designed to be the "easiest" items to pass on the questionnaire), then the 4-month questionnaire was administered.

3) If the caregiver's responses indicated a child passed all 6 items on the 6- month questionnaire, the caregiver was advanced to the 8-month questionnaire.

4) A caregiver advanced to subsequent questionnaires until their responses indicated a child did not pass any items of a given questionnaire.

5) For children without noticeable developmental delay, interviewers were instructed to start the caregiver on the 12-month questionnaire and progress to the questionnaire appropriate for the next highest or lowest age level, as described above.

We took several steps to account for the fact that the questionnaire used to assess a child might have been designed to assess a younger child. First, we calculated an ASQ z-score for each of the

5 developmental domains the instruments are designed to assess, using a child's score on the questionnaire administered. We compared this z-score to the mean and SD of Brazilian children who were assessed with that same questionnaire (Filgueiras, Pires, Maissonette, & Landeira-Fernandez, 2013). We then used these ASQ z-scores to obtain a DQ and calculated the z-score for the DQ for each child on each domain. These steps were taken to calculate a final DQ that reflected the relation of the age at which the child was functioning (as reported by their caregiver) to their biological age. Further details are provided below.

Developmental Delay

We characterized developmental delay by: 1) estimating the DQ score given the ASQ-3 z-score, 2) adjusting the DQ score based on the child's age and test-appropriate age, and 3) converting the adjusted DQ score to a DQ z-score. This procedure is outlined below.

Developmental Quotient (DQ) Estimation

ASQ Z-Score

The ASQz score is defined as:

$$\frac{ASQ - \mu^{ASQ}}{\sigma_{ASQ}} \tag{1}$$

Where μ^{ASQ} and σ_{ASQ} characterize the distribution in the Brazilian child population, by age class, and ASQ is the child's score from the ASQ-3 questionnaires administered for the ZODIAC Investigation.

We then converted the ASQ^z to a DQ to quantify developmental delay (DD). This required a conversion factor, 10/9, to shift the ASQ^z to the DQ scale, see equation (4). The conversion factor is multiplied by the ASQ^z and the product is multiplied by σ_{DQ} and then added to μ^{DQ}.

This value is then adjusted by the ratio of Age^e/Age^a, where Age^e is the administered test equivalent age and Age^a is the child's age at the time of the administered test.

$$\widehat{DQ} = \left[\mu^{DQ} + \sigma_{DQ} \left(\frac{10}{9}\right) ASQ^z\right]\left(\frac{Age^e}{Age^a}\right) \tag{2}$$

Developmental Quotient Z-Score

The resulting DQ estimate is then used to calculate a DQ^z, where by definition, $\mu^{DQ} = 100$, and $\sigma^{DQ} = 15$.

$$DQ^z = \frac{DQ - \mu^{DQ}}{\sigma_{DQ}} \tag{3}$$

ASQ^z to DQ^z Conversion Factor

The figure below provides the information to estimate the ASQ^z to DQ^z conversion factor. Note in Figure 1 that when $ASQ^z = -1.5$ the corresponding DD value is 25%. By definition, DQ = 100 − DD = 75. Using equation (3) we estimate DQ^z given DQ = 75, i.e.,

$$DQ^z = \frac{75-100}{15} = -\frac{5}{3} \tag{4}$$

Figure. ASQ Distribution and Percent Delay Estimates

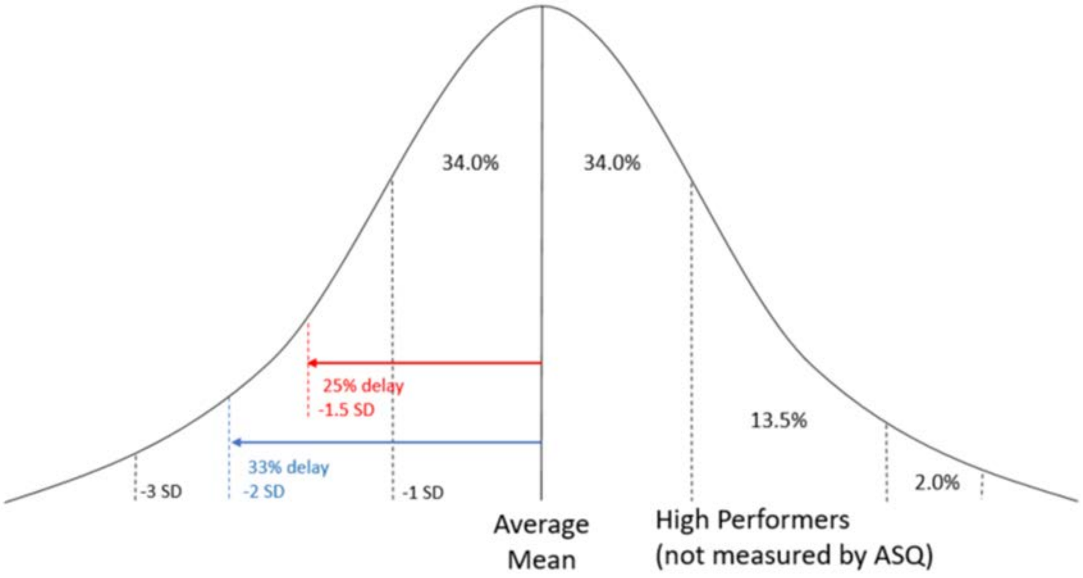

Note: SD, Standard Deviation; Y-axis represents the percentage of children in the ASQ-3 normative population with a score in each standard deviation range (Unpublished figure courtesy of a developer of the ASQ-3 instruments, Jane Squires, Ph.D., Professor, College of Education, Center on Human Development, University of Oregon.)
